# Assessing nutritional composition and ingredients of packaged foods in Brazil: an in-store census method for creating a comprehensive food label database

**DOI:** 10.3389/fnut.2025.1568089

**Published:** 2025-06-18

**Authors:** Mariana Vieira dos Santos Kraemer, Ana Carolina Fernandes, Maria Cecília Cury Chaddad, Tailane Scapin, Beatriz Vasconcellos de Barros, Elisa Milano, Marina Padovan, Paula Lazzarin Uggioni, Greyce Luci Bernardo, Neha Khandpur, Gastón Ares, Rossana Pacheco da Costa Proença

**Affiliations:** ^1^Nutrition in Foodservice Research Centre, Nutrition Postgraduate Program, Federal University of Santa Catarina, Florianópolis, Brazil; ^2^Movimento Põe no Rótulo, São Paulo, Brazil; ^3^Global Centre for Preventive Health and Nutrition (GLOBE), Institute for Health Transformation, School of Health and Social Development, Deakin University, Burwood, VIC, Australia; ^4^Division of Human Nutrition and Health, Wageningen University, Wageningen, Netherlands; ^5^Sensometrics and Consumer Science, Instituto Polo Tecnológico de Pando, Facultad de Química, Universidad de la República, Montevideo, Uruguay

**Keywords:** nutrition labeling, ingredient list, nutrition information, ultra-processed foods, methods, supermarkets

## Abstract

**Background:**

The consumption of ultra-processed packaged foods has surged worldwide with important health implications. It is pertinent to study the composition of packaged foods through information provided on labels. However, there is limited methodological discussion in the field. This study aimed at discussing methodological evolution and challenges of in-store census methods for assessing the composition of packaged foods, and characterizing a Brazilian food label database.

**Methods:**

The first Brazilian food label database reported in the scientific literature, based on data of in-store census method, was created in 2010 by the Nutrition in Foodservice Research Centre (NUPPRE at the Federal University of Santa Catarina). The in-store census method involves collecting primary data directly from the labels of packaged foods available for sale through retail food outlets. In 2020, the in-store census was carried out in partnership with the FoodSwitch Program. The NUPPRE/FoodSwitch Brazil 2020 database was developed in four steps: pre-data collection, data collection, data tabulation, and database construction and processing. The database was characterized by calculating the prevalence of foods per food group and foods that declared mandatory nutrition and health information on food labels according to Brazilian regulation.

**Results:**

The nutritional profile and ingredients of packaged foods was obtained from four food label censuses (2010, 2011, 2013 and 2020), supporting the Brazilian government on food labeling regulations and public policies. The experience prompted reflections about the methodological aspects of food label studies, and enabled improvements to the research process, such as a more accurate data collection, the inclusion of all packaged foods and beverages available for sale in the supermarket and the inclusion of more variables to the analysis. It is noteworthy the relevance of building nationwide food labeling databases. However, there are important challenges regarding the costs and efforts needed to maintain and update the data, especially in continental countries such as Brazil. The NUPPRE/FoodSwitch Brazil 2020 database consists of 7,828 packaged foods, 94% of the sampled brands sold nationwide. Most foods presented the mandatory information according to Brazilian regulation.

**Conclusion:**

This study proposed a series of methodological procedures to be carefully considered, designed, and executed during planning, data collection, data tabulation, and database processing. Greater rigor and detail are needed in the methods section of scientific articles, to aid replication.

## 1 Background

Over the past decade, the consumption of processed and ultra-processed foods has surged worldwide and these products have partially replaced fresh unprocessed foods with important public health implications (1). In particular, consumption of ultra-processed foods has been associated with the development of several diseases, such as cardiovascular diseases, type 2 diabetes, obesity, numerous types of cancers, mental disorders, as well as with an increased risk of all-cause mortality (2, 3). The proposed mechanisms to explain these associations are related to the processing-altered composition of ultra-processed products: high content of sugars, saturated fat, trans fat, and/or sodium, and the presence of food additives, as well as neoformed compounds, and contamination with contact materials (3–5).

In this context, monitoring the composition of the processed and ultra-processed products available in the marketplace is relevant to inform the design, implementation, monitoring, and evaluation of public health interventions aimed at improving diet quality. Food composition data are also relevant for clinical and epidemiological research, particularly in relation to the health effects of ultra-processed foods (6).

Food composition data are available on food labels as manufacturers are mandated to include the list of ingredients and nutrient declarations in most countries (7). Therefore, food labels can be used to monitor the composition of processed and ultra-processed products (8), to the extent that they are reported transparently, accurately and consistently by food manufacturers. Any informational limitation of the nutritional labels may also be reflected in the databases constructed from them. The main advantage of this approach is that large databases can be compiled in short-time frames at relative low cost compared to chemical analyses. Food labels are also the key source of information for consumers, enabling them to access detailed information about the nutritional and ingredient composition of packaged foods (6).

Databases containing food labeling information have been developed worldwide, which differ in data collection procedures and data quality control, among other aspects. Some publicly available databases (e.g., Open Food Facts) rely on crowdsourcing, where food label photographs are submitted by users (9). Other labeling databases contain information provided directly by food manufacturers. Examples include the USDA’s *FoodData Central* (10) and the Mintel database (11). However, the completeness and accuracy of the data may not always be guaranteed (6, 12). In addition, some of these databases are behind paywalls (e.g., Mintel), which may limit their accessibility.

Label information can also be collected from the websites of food manufacturers and retailers (13). However, these websites usually include limited information. A recent study reported that food composition information was frequently unavailable on supermarket websites in Australia, such as allergen information, the nutrition facts panel, and the ingredients list (13). Food composition databases have also been created by researchers by collecting primary data directly from the labels of packaged foods available for sale through retail food outlets (12, 14, 15). Data collection is carried out in person at supermarkets or other food outlets, by collecting information from the labels of all the available products to generate a comprehensive database. In the current study, this strategy is referred to as the in-store census method.

Databases have been generated using in-store census worldwide. Examples include the FoodSwitch database from Australia (16, 17), the Food Label Information Program (FLIP), from Canada (18), and the Composition and Labeling Information System (CLAS), from Slovenia (6). In Brazil, the Nutrition in Foodservice Research Centre (NUPPRE) at the Federal University of Santa Catarina (UFSC) has been performing in-store census of food labels since 2010.

Despite the importance of monitoring food composition using label information, the great majority of the articles published with food labeling data do not discuss the methodological aspects related to the construction of the database, which may directly impact the quality of the data. Few studies have been published describing in detail the procedures for collecting, tabulating, and constructing databases from food label information (6, 12, 14, 15). In view of these gaps, this study aimed at: (i) discussing methodological evolution and challenges of in-store census methods for assessing the composition of packaged foods, and (ii) describing and characterizing the NUPPRE/FoodSwitch Brazil 2020 food label database.

### 1.1 The Brazilian food labeling regulatory framework

The Brazilian Health Regulatory Agency determines the criteria for the declaration of food labeling information through several regulations. Brazilian Resolution No. 259/2002 establishes general rules for the labeling of packaged foods, requiring the declaration of mandatory information on food labels, such as the ingredient list. The only exception to this requirement applies to foods composed of a single ingredient, such as sugar, coffee, and salt, which do not need to comply with those requirements (19).

Another legal instrument, Resolution No. 360/2003, addresses the nutrition and health aspects of labeling, and made nutrition labeling mandatory for all packaged foods. Nutrition labeling includes information presented on the nutrition facts panel (mandatory) and nutrition claims (voluntary). The following items must be declared on the nutrition facts panel, accompanied by their respective quantities per serving: energy value (kcal and kJ), carbohydrates (g), proteins (g), total fat (g), saturated fat (g), trans fat (g), dietary fiber (g), and sodium (mg). The declaration of vitamins and other minerals is optional if the product contains 5% or more of the recommended daily intake per serving, whereas it should be mandatory if the front of the package contains any claim about these nutrients (20). Additionally, this resolution did not apply to the following foods: alcoholic beverages; food additives and processing aids; spices; mineral waters; vinegars; salt; coffee, yerba mate, tea, and other herbs without additional ingredients; and fresh, chilled, and frozen meats, fruits, and vegetables. Therefore, data on these foods were not collected in 2010, 2011 and 2013.

Three other regulations concerning the declaration of nutrition and health information related to gluten, allergens, and lactose were in force at the time of data collection. All packaged foods with labels must contain the warning “contains gluten” or “does not contain gluten,” as appropriate (21). Furthermore, all packaged foods with labels must declare the presence of the following allergens: wheat, rye, barley, oat and oat hybrids, crustaceans, eggs, fish, peanut, soybean, milk from any species of mammalian animals, almond, hazelnut, cashew nut, Brazil nut, macadamia nut, pecan nut, pistachio, pine nut, chestnut, and natural latex (22). Finally, regarding lactose, the applicable resolution requires the declaration of lactose presence for all packaged foods with labels that contain more than 100 mg of lactose per 100 g or 100 mL (23).

It should be noted that all these regulations were in force by the time of 2010, 2011, 2013 and 2020 data collections, therefore, being applied uniformly across the entire studied periods. Most of these mandatory requirements are still in effect, though they are now regulated by a recently approved resolution (24), which consolidated the general labeling legislation. Also, a new regulation regarding nutrition labeling was approved in October 2020 (25), significantly altering the regulatory framework. However, the impacts of this change are not within the scope of this investigation, given that the regulation was not in force by the time of the data collections.

### 1.2 Historical and methodological evolution of the in-store food label data collection in Brazil: an overview of the NUPPRE/UFSC census method for creating comprehensive food labeling databases

The first Brazilian food label database reported in the scientific literature, based on data collected using an in-store census method, was created in 2010 by NUPPRE/UFSC (26). Over time, the methodological aspects were refined to enhance the validity and reliability of the database. Table 1 presents the main methodological aspects of the label census method developed by NUPPRE/UFSC (2010, 2011, 2013) and NUPPRE/FoodSwitch Brazil 2020.

**Table 1:** Description of the four in-store census methods (2010, 2011, 2013, and 2020) used to develop comprehensive food labeling databases in Brazil.

Methodological aspect	In-store label census			
	NUPPRE Brazil 2010 (*N* = 2,327)	NUPPRE Brazil 2011 (*N* = 4,286)	NUPPRE Brazil 2013 (*N* = 5,620)	NUPPRE/FoodSwitch Brazil 2020 (*N* = 7,828)
Study site				
Large supermarket chain^§^	X	X	X	X
Medium-size store	X			
Large-size store		X	X	X
Trained data collectors	X	X	X	X
Data collection instrument				
Paper form	X	X		
Electronic form			X	
Smartphone app with a photo capture feature				X
Information collected from food labels				
Product identification	X	X	X	X
Trans fat information (quantity and claims)	X			
Sodium information (quantity and claims)		X		
All nutrient information for all nutrients available (quantity and claims)			X	X
All claims related to trans fat	X			
All claims related to sodium		X		
All claims presented in the packaged (nutrition claims, health claims and others)			X	X
Trans fat ingredients	X			
Sodium-containing ingredients (salt and sodium-based ingredients)		X		
All ingredients (food ingredients and food additives)			X	X
Diet/light claims		X		X
Marketing strategies targeting children		X	X	X
Transgenic (GMO) symbol			X	X
Additional information displayed on the label			X	X
Inclusion criteria				
Foods that may contain trans fat	X			
Foods that may contain sodium		X		
Foods within the scope of Brazilian nutritional labeling legislation^**^ (except foods intended for infants and young children)	X	X	X	
All packaged foods and beverages available for sale at the supermarket				X
Data collection procedures				
Data collection begins after supermarket authorization	X	X	X	X
Paper forms	X	X		
Electronic forms			X	
All sides of the package of all packaged foods available for sale at the supermarket by the time of data collection were photographed			X	X
Data tabulation				
Transcription of collected information into a Microsoft Excel spreadsheet	X	X		
Transcription of ingredients lists into a Microsoft Excel spreadsheet			X	
Information collected in the electronic form transferred directly to Microsoft Excel spreadsheets			X	
Transcription of collected information into a monitoring database				X
Database processing				
Exclusion of duplicated products	X	X	X	X
Data transferred from the monitoring database (FoodSwitch program) to Microsoft Excel				X
Quality control: verification of tabulated data in 10% of the database	X	X	X	
Quality control: verification of tabulated data in 20% of the database and in each study based on variables of interest				X
Focus of data analysis				
Trans fat	X		X	X
Trans fat substitutes			X	X
Serving sizes and household measures	X			
Sodium		X		
Foods targeted at children or consumed by children		X	X	X
Added sugars			X	X
Added sugars in foods targeted at children				X
Free sugars from fruits				X
Sweeteners (food additives)			X	X
Homemade, traditional, and similar claims			X	X
Whole grain claims			X	
Claims of functional and health properties				X
Claims of functional and health properties in foods targeted at children				X
Genetically modified organisms			X	
Vitamins and minerals in foods targeted at children			X	
Food additives				X
Food additives in foods targeted at children				X

^§^According to the revenue ranking annually published by the Brazilian Association of Supermarkets ^*^Brazilian Health Regulatory Agency (20); ^**^Brazilian Health Regulatory Agency (30).

The first data collection, in 2010, took place in the context of a study on the declaration of trans fat, serving sizes, and household measures on packaged food labels. Food label data were gathered at a medium-sized supermarket store belonging to a large Brazilian chain. This supermarket chain remains one of the leading chains in Brazil, according to the revenue ranking published by the Brazilian Association of Supermarkets (27).

The data were recorded on a paper-based form. Information was manually copied from labels at the supermarket by trained data collectors. The sample included all foods likely to contain trans fat that were available at the supermarket at the time of data collection. Variations of the same type of food product (different flavors and packaging sizes) were counted as distinct items, as it was observed that products often had different characteristics and compositions depending on the size and type of packaging. Foods outside the scope of nutritional labeling legislation (20) were not included in data collection. Additionally, foods intended for infants and young children were also excluded, as they were regulated by specific legislation (28). Subsequently, the data were transcribed into Microsoft Excel spreadsheets for analysis. The following information was collected: trade name, brand, manufacturer, country of origin, product type (e.g., cookies, milk drink, and chocolate), flavor, price, package weight, presence/absence and order of declaration of trans fat or ingredients likely to contain trans fat in the ingredients list, presence of nutrition information on trans fat on the nutrition facts panel, serving size, household measure, and claims related to the absence of trans fat.

In 2011, a new data collection was carried out at a large supermarket store of the same chain chosen in the previous year. Methodological procedures for data collection and tabulation were also the same but focused on the analysis of sodium declaration in packaged foods. The sample included foods that could contain sodium, were covered by the applicable nutrition labeling legislation (20), and were available for sale at the supermarket. The following information was collected from product labels: trade name, brand, manufacturer, country of origin, product type, flavor, price, package weight, presence/absence and order of declaration of added sodium (salt and sodium-containing food additives) in the ingredients list, nutrition information on foods containing added sodium, sodium declaration on the nutrition facts panel, serving size, household measure, sodium-related claims, and claims targeted at children.

In 2013, NUPPRE/UFSC conducted a third in-store census of food labels. Data collection was performed at the same supermarket store sampled in 2011. The data were recorded in-store by trained data collectors using tablets and an electronic form (EpiCollect Plus®), based on the previously used paper-based form. For this data collection, all food products also had their packages photographed. Subsequently, the information collected on the electronic form was exported to Microsoft Excel. The photos were used to extract data from the ingredients lists of each product, and this information was tabulated in Microsoft Excel. The sample included all foods available for sale at the supermarket and covered by applicable legislation (20). The information collected from product labels included trade name, brand, manufacturer, country of origin, product type, flavor, price, package weight, regulated nutrition claims (29), nutrition facts panel information (serving size, household measure, total energy value, carbohydrates, proteins, total fat, saturated fat, trans fat, fiber, sodium, vitamins, and minerals), and ingredients list. As in the two previous data collections, product variations (different flavors and package sizes) were considered distinct items, and infant and children’s foods were excluded.

For the first time, in 2020, data collection was carried out in partnership with FoodSwitch. The sampled supermarket store was the same site as 2011 and 2013 data collections. A database was created using data on the nutritional composition of all packaged foods and beverages available for sale at the time of data collection.

Information on the nutritional profile and ingredients of packaged foods was obtained from the four food label censuses conducted by our research group. Such data were utilized for various analyses related to the nutritional and ingredient composition of packaged foods, including cross-sectional and longitudinal analyses, comparisons, and data monitoring. The studies were carried out in the form of postdoctoral fellowships, doctoral theses, master’s dissertations, scientific initiation projects, and undergraduate capstone projects with Pan American Health Organization (PAHO), Brazilian Health Regulatory Agency (ANVISA) and Brazilian national agencies for research support (CAPES and CNPq) grants (Supplementary Table S1). Additionally, our research group has been supporting ANVISA and the Brazilian Ministry of Health in important actions, as recognition and result of the work developed over the four NUPPRE in-store censuses (2010, 2011, 2013, and 2020), assisting the development and reformulation of public policies in food, nutrition, and health. In this perspective, efforts have been made toward reforming national legislation regarding general and nutritional food labeling, eliminating trans fat, defining the appropriate use for the term “whole” in cereal- and pseudocereal-based foods, establishing quality standards for oils and fats, and engaging in discussions about food additives, sweeteners, and added sugars.

With the experience gathered after every data collection, it was possible to improve the methodological procedures. The main methodological differences between the four censuses (2010, 2011, 2013, 2020) refer to (i) the type of foods included in data collection, (ii) the information retrieved from food labels, and (iii) data collection instruments and techniques.

With each new data collection, efforts were made to expand the range of packaged foods analyzed. In the first two data collections, only foods possibly containing trans fat or sodium were analyzed. In the third data collection, the aim was to gather data from all packaged foods covered by Brazilian nutrition labeling legislation. In 2020, all labeled foods and beverages marketed by the supermarket were censused, including those outside the scope of nutritional labeling regulations, such as alcoholic beverages, mineral waters, vinegars, salt, coffee, meats, fresh fruits and vegetables, and other items described above. Thus, data were collected from all labeled foods available for sale in the supermarket at the time of data collection.

From 2013 onwards data collection no longer occurred using paper forms, but in a mixed manner: an electronic form was filled, and photographs were taken of food products. In 2020, data collection was carried out entirely by taking photographs of food labels. This method helped reduce the risk of bias related to errors in data collection and allowed further expanding the amount of information collected. Thus, in addition to the data tabulated in 2013, in 2020, it was possible to gather more information available in food labeling, such as allergens, gluten, and lactose.

Regarding claims (nutrition, health, and others), those related to trans fat were included in the 2010 database; the claims related to salt, and sodium were included in the 2011 database; and the nutrition claims as regulated by Brazilian legislation (29) were included in the 2013 database. In 2020, although front-of-pack claims were available in the photos taken during data collection, this information was not tabulated and, consequently, was not included in the database. This decision was taken because of the wide diversity of claims displayed on the front of the package by manufacturers. Future data tabulation by researchers with expertise in the subject could enhance precision and accuracy, reducing the potential for errors. For instance, the tabulation of claims linked to marketing strategies targeting children was handled by researchers working on the theme, allowing for the application of more specific and accurate criteria to identify such claims.

Concerning data collection instruments, the use of an electronic form in 2013 made it possible to export part of the information directly to a Microsoft Excel spreadsheet; thus, only the ingredients list was transcribed. In 2020, an app was used to take photographs of food labels, increasing the speed of data collection. This method helped reduce the risk of bias related to errors in data collection.

All collections were carried out in the same supermarket chain, which is among the largest supermarket chains in Brazil according to annual revenue (27). In the first census, a smaller store was selected due to the limited technical capacity of the team and the pioneering nature of the study, ensuring quality and continuity in data collection. As the team’s technical skills and experience enhanced, the second data collection was conducted in a larger store. According to data from the supermarket chain, the store chosen for the 2011, 2013, and 2020 censuses is the largest with regard to size, number of products, and number of brands. Given the large store size, it was possible to collect information from a greater diversity of products and brands.

## 2 A step-by-step approach to building a food label composition database: description of the NUPPRE/FoodSwitch 2020 in-store census

**Figure 1:**
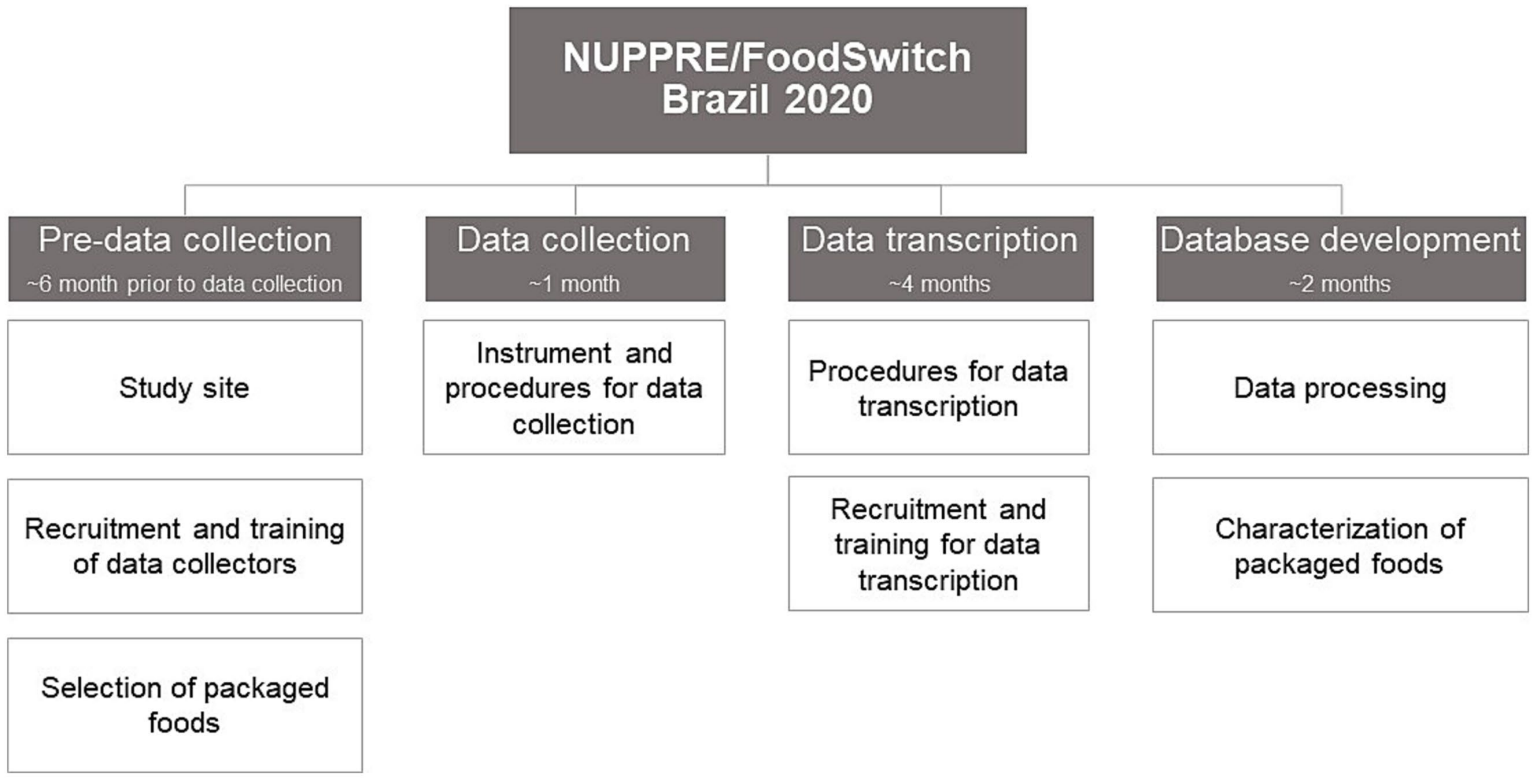
Stages of development of the NUPPRE/FoodSwitch Brazil 2020 database.

The development of the NUPPRE/FoodSwitch Brazil 2020 database comprised four distinct and consecutive steps: pre-data collection procedures, data collection, data tabulation, and database construction and processing. Each step was carried out in stages, as depicted in Figure 1.

### 2.1 Pre-data collection procedures

#### 2.1.1 Study site selection

The study was conducted during November 2020 in a large supermarket outlet in Brazil. The outlet was chosen intentionally, to enable data collection with the available financial and human resources. In addition, the outlet was selected because it belonged to one of the 15 largest supermarket chains in Brazil according to annual revenue (27) and was the largest supermarket outlet in the Brazilian state where the study was conducted (Santa Catarina). The supermarket manager provided consent for data collection.

#### 2.1.2 Recruitment and training of data collectors

Ten data collectors were recruited among graduate and undergraduate students in Nutrition & Dietetics at UFSC. All data collectors received theoretical-practical training, offered in English by researchers from the Australian FoodSwitch program. The training session was conducted virtually and covered the configuration and operation of the data collection instrument, as well as practical and technical aspects of data collection in supermarkets. The research coordinators offered a reinforcement training session in Portuguese, the native language of data collectors. Additionally, the data collectors received a document outlining the data collection protocol. The protocol included a detailed explanation of how to use the data collection instrument, encompassing operations such as filling in identification data, scanning barcodes, taking and saving photos, and completing the data collection session.

A pre-test of the data collection process was carried out to identify potential errors in operating the instrument and to ensure that photos of food packaging were taken correctly. In the pre-test, data collectors were instructed to use the data collection instrument to take photos of six distinct food labels, following the procedures taught during training. Then, the Australian FoodSwitch team provided feedback on the quality and legibility of photographs and collected information.

#### 2.1.3 Inclusion criteria

Information was collected from the labels of all packaged foods available for sale at the time of data collection that met the following inclusion criteria:

- All foods that are marketed and packaged in the absence of the customer and ready for consumer purchase, according to Resolution No. 259/2002 (19), which specifies labeling requirements for packaged foods.
- Specific foods for infants and young children, as defined by Law No. 11265/2006. These products include formulas for infants, follow-on formulas for infants and young children; fluid, powdered, and modified milk products and similar products of plant origin; transition and cereal-based foods indicated for infants and/or young children; foods or beverages, whether milk-based or not, suitable for infants and young children (28).
- Alcoholic beverages and mineral waters.

Foods with different barcode numbers were treated as distinct products. Therefore, all variations of a food product (different flavors and package sizes) were sampled, as products can have distinct characteristics and composition depending on packaging size and type. Unpackaged fresh fruits, vegetables, meats, breads and bakery products sold without a label were not surveyed.

### 2.2 Data collection

**Figure 2:**
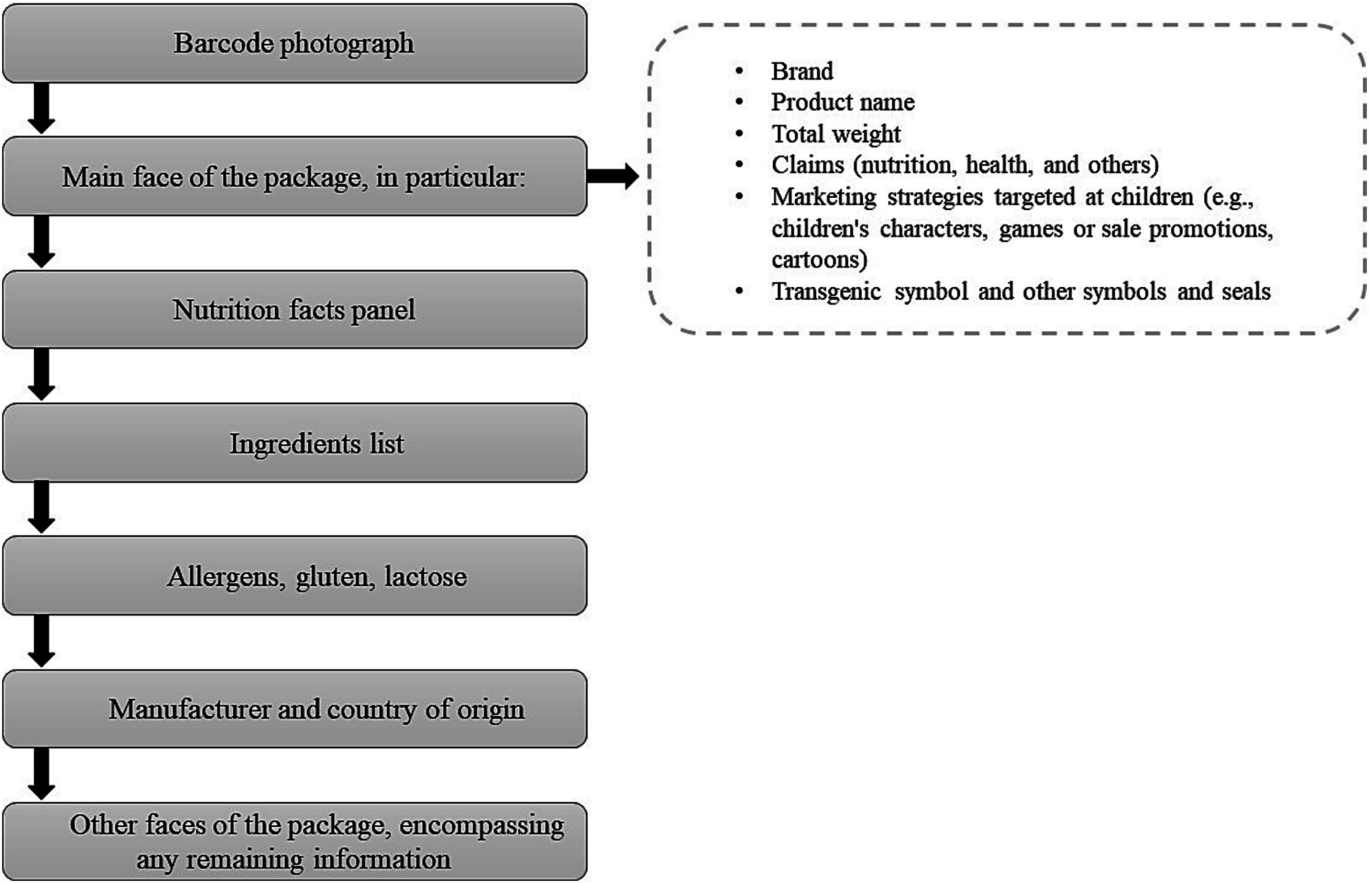
Information retrieved from photographs taken during data collection for the creation of the NUPPRE/FoodSwitch Brazil 2020 database, in the order in which they were recorded.

Labels were photographed using a mobile phone application developed by The George Institute’s FoodSwitch program, Australia (16, 17). The application was adapted for collecting data from Brazilian food labels on iOS and Android smartphones. The app enabled data collectors to scan the barcodes of each packaged food for identification and then take photos of the information displayed on the labels. Figure 2 shows the information retrieved from photographs, presented in the order in which they were recorded.

If necessary, more than one photo could be taken to capture all required information. For example, if it was not possible to fit the entire nutrition facts panel into a single photo, collectors could take as many photos as needed. Once all the information had been retrieved, the data collection for that product was completed, and the next item was surveyed by scanning the barcode.

As in the previous years (2010, 2011, and 2013), a coordinator was present during data collection in 2020 and assisted data collectors in case of difficulties. On each day, the coordinator informed the data collectors which supermarket sector would be surveyed and instructed that data from all food products available in that sector should preferably be collected on the same day. All foods available for sale at the time of data collection were surveyed.

At the end of data collection, the photographs were uploaded to the FoodSwitch system. These photos were later made available for data tabulation.

### 2.3 Data tabulation

#### 2.3.1 Data tabulation procedures

Tabulation consisted of transcribing the information contained in food label photos to a monitoring database developed by FoodSwitch (The George Institute’s Food and Beverage Information Content Management System—FBI CMS). The monitoring database is an online platform where photos are organized by food products, based on their barcode numbers. Next to each photo, there are fields for transcribing the details shown in the images.

The following information was tabulated by the researchers, in Portuguese: manufacturer, brand, product name, total package weight, and nutrition facts panel information (serving size, household measure, macro- and micronutrient contents, ingredient list, list of allergens, contains/does not contain gluten, alcohol content).

#### 2.3.2 Recruitment and training of data tabulators

As for data collection, 10 data tabulators were recruited among graduate and undergraduate students in nutrition at UFSC. All data tabulators participated in a 2 h training session, provided virtually in English by researchers from the Australian FoodSwitch program. The training session addressed practical questions about the monitoring database, including which information should be entered in each field. The research coordinator offered a reinforcement training session in Portuguese, the native language of data tabulators. This reinforcement session lasted about 1 h and was aimed at clarifying doubts and highlighting key points of data tabulation. Additionally, data tabulators received a step-by-step protocol. The protocol provided a detailed explanation of how to use the monitoring database and indicated where each piece of information should be entered.

### 2.4 Database development and processing

After tabulation, the data were exported to a Microsoft Excel® spreadsheet, creating the NUPPRE/FoodSwitch Brazil 2020 database. The database was made available on a remote desktop, with individual access granted to each researcher.

In a preliminary treatment step, two different researchers reviewed the database for duplicate products, which were identified in the Excel spreadsheet by their barcode numbers. When the same food was tagged as a duplicate by the two researchers, one of the duplicate entries was excluded from the database. Of note, the only criterion for excluding food products from the database was the presence of duplicate entries. A lack of nutrition, ingredient, or health-related information was not adopted as an exclusion criterion.

The next step involved the food products classification into groups and subgroups, according to the Brazilian nutrition labeling legislation in effect at the time (20). Additional groups were created for foods not covered by this classification, namely Baby and infant foods; Mineral waters; Non-sugar sweeteners, colorings, flavorings, raising agents, and yeasts; Tea, herbs, and coffee; Vinegar and salt; and Supplements.

Quality control has been carried out in 20% of the database, as well as in all studies conducted by NUPPRE researchers using this database, focusing on specific variables of interest. For instance, a study analyzing trans fat content in packaged foods would verify fat information for a portion of the foods entered in the database, compared to the information collected manually or through the photo, proposing corrections as needed.

### 2.5 Database characterization

The NUPPRE/FoodSwitch Brazil 2020 database was characterized by calculating the number of food products per food group, stratified as defined by the applicable nutrition labeling legislation (30).

The declaration of mandatory nutrition and health information on food labels was assessed based on the following criteria: (i) presence of the nutrition facts panel (20), (ii) presence of the ingredients list (19), (iii) presence of allergen information (22), (iv) presence of the “contains/does not contain gluten” warning (21), and (v) presence of lactose information (23).

## 3 Characterization of foods composing the NUPPRE/FoodSwitch Brazil 2020 database

The NUPPRE/FoodSwitch Brazil 2020 database includes 7,828 packaged products of 1,035 different brands, 94% of which were sold nationwide. Table 2 shows the frequencies of packaged foods and the mandatory components of food labels related to nutrition and health information, stratified by food group. Most food items belong to the non-alcoholic beverages, sweets, and confectionery group (25%). Sweet biscuits, chocolates, non-alcoholic beverages, and savory snacks are the most frequent foods in this group, corresponding to 50% of the items in the group. The second most prevalent group was cereals, vegetables, and tubers (14%), in particular salted biscuits, breads, and pasta (42%).

**Table 2:** Characteristics of food items composing the NUPPRE/FoodSwitch Brazil 2020 database, stratified by food group and presence of mandatory items on food labels.

Food group	Frequency		Display of mandatory items on food labels^*^									
			Nutrition facts panel		Ingredient list		Allergen information		Gluten information		Lactose information	
	*n*	%	*n*	%	*n*	%	*n*	%	*n*	%	*n*	%
Cereals, legumes, and tubers^§^	1,092	14	1,086	99	1,008	92	897	82	1,073	98	80	7
Vegetables^§^	366	5	355	97	313	86	40	11	349	95	2	1
Fruits^§^	311	4	308	99	248	80	45	14	304	98	0	0
Milk and dairy^§^	804	10	790	98	799	99	765	95	798	99	418	52
Meat, pork, poultry, and seafood^§^	736	9	713	97	602	82	397	54	722	98	43	6
Oils and fats^§^	404	5	400	99	386	96	249	62	396	98	54	13
Non-alcoholic beverages, sweets, and confectionery^§^	1,966	25	1,935	98	1,945	99	1,483	75	1,941	99	510	26
Sauces and ready-to-eat dishes^§^	580	7	447	77	562	97	334	58	569	98	81	14
Baby and infant foods	72	1	72	100	72	100	53	74	72	100	12	17
Alcoholic beverages	941	12	21	2	887	94	275	29	885	94	1	0
Mineral waters	64	1	34	53	0	0	0	0	56	87	0	0
Non-sugar sweeteners, colorings, flavorings, raising agents, and yeasts	67	1	42	63	67	100	22	33	66	99	0	0
Tea, herbs, and coffee	307	4	66	21	190	62	18	6	300	98	0	0
Vinegar and salt	62	1	29	47	58	94	0	0	61	98	0	0
Food supplements	56	1	54	96	56	100	31	55	54	96	14	25
Total	7,828	100%	6,352	81%	7,193	92%	4,609	59%	7,646	98%	1,215	16%

^§^Food groups were classified according to the Brazilian legislation on nutrition labeling (30) in force during data collection (November 2020). ^*^Mandatory information related to nutrition or health information, according to Brazilian legislation on food labeling (19–23) in force during data collection (November 2020). The absence of mandatory information does not necessarily mean a non-compliance with Brazilian legislation, considering that there are exceptions depending on the food category.

Most food items presented the mandatory information on the presence of gluten (98%), ingredient list (92%), nutrition facts panel (81%), and allergens (59%) (Table 2). It was notable that all items of the baby and infant food group (which includes infant formulas and infant cereals) had labels containing an ingredient list, nutrition facts panel, and gluten information. Information on lactose was the least frequent on the food labels of all groups.

It was found that most foods complied with national regulations regarding mandatory health and nutrition information. As expected, information on the presence of lactose was the least frequent, as it applies to a smaller universe of foods, that is, only those with more than 100 mL of lactose in 100 g or 100 mL of food (23). Gluten must be declared on all packaged foods and beverages (21). The absence of other mandatory items on food labels does not necessarily constitute non-compliance with Brazilian legislation, as there are exceptions to their presence on labels. For example, the presence of allergens must be declared in foods that contain these substances or are at risk for unintentional contamination. Thus, foods that do not contain allergens and do not pose a risk of contamination are not required to include allergen statements on the label. Similarly, there are specific rules for lactose, ingredients lists, and nutrition facts panels, as explained in the Methods section. Considering the public health relevance of allergens and lactose declaration on food labeling, it is important to develop studies aiming to analyze the labeling of these components in order to monitor compliance with specific regulations and assess whether accurate information is being provided to consumers.

## 4 Challenges and lessons learned during the application of the in-store census method

The experience gained over the four data collections by NUPPRE/UFSC prompted reflections about the methodological aspects of food label studies, which are still incipient in scientific literature.

During the planning of this study, we highlight the importance of previously establishing clear criteria on which foods would compose the database and what information should be retrieved from food labels. Factors such as financial resources, human resources, available time, and technical capacity to conduct the studies and work with the data need to be considered. Another important factor is defining where data are to be collected. The supermarket, or other points of sale, should be chosen based on the objectives of the study, establishing clear criteria and seeking the place that best suits the context. It is important to conduct data collections that cover the greatest diversity of foods, brands, and manufacturers possible, thereby addressing data on products that are available to a greater number of people.

Collecting data from food labels through photographs is faster and more accurate compared to paper or electronic forms, when the aim is to collect all information available on food labels. A smartphone application that takes photographs and sends them to data clouds according to the respective barcode number was used here and in previous research conducted in other countries (16–18, 31). Automated tools that enhance the speed of data collection also contribute to avoiding errors generally caused by data collectors. It is important to adequately manage in-store data collection by organizing the team and conducting prior training so that photographs are taken correctly, and products are not missed. For errors and unforeseen events to be avoided during data collection, it is important to carry out a pre-test of the collection instrument, as well as a pilot test of data collection. These procedures allow improving the use of instruments and collection techniques.

In all data collections carried out by NUPPRE, foods with varying packaging sizes were considered as different products. This criterion was adopted because, since the first collection, it was observed that some products have different characteristics and compositions according to packaging size and type, in addition to having different barcode numbers. This criterion is considered a relevant methodological measure, ensuring that all foods sold at the time of data collection are analyzed. An additional methodological measure to ensure the analysis of all food items sold in the supermarket was to have a coordinator present every day. This daily monitoring made it possible to help data collectors, minimizing possible errors and potential biases arising from failures. In addition, the coordinator managed and monitored the evolution of data collection, ensuring that all sectors of the supermarket and all food items were contemplated and photographed.

Studies adopting an in-store census method (32, 33) generally analyze a smaller number of food items than studies using data from online searches or existing databases based on crowdsourcing or information from food manufacturers (14, 34). These differences may be attributed to the characteristics of the method, in particular, the need for in-person visits to food sales locations. However, when using an in-store census method, it is possible to clearly determine the criteria for including foods in the database, that is, all those sold at the time of data collection. Furthermore, this strategy encompasses all foods available to consumers at the time of purchase, working with data collected in a real environment.

Transcription of the information on food labels is the costliest stage of the study in terms of time and human resources. The use of technology to extract data from photographs is still a challenge, representing an important future perspective for studies in the area. Optical Character Recognition (OCR) software can transcribe data from images; however, there are still barriers to its use for scientific research. Additionally, the legibility of labels is not always adequate, impairing automatic transcription of information. To date, one study used an artificial intelligence tool for the extraction of symbols from food labels (35), and one study used artificial intelligence to automatically extract written data (nutrition information panel and list of ingredients) from photographs (14). However, the photographs were captured from websites and the authors underscored the need for human validation to determine the accuracy of the extracted information (14).

We highlight two relevant methodological precautions regarding the treatment of data in food label databases. The first is the assessment and exclusion of duplicate foods in databases. Although the data collection app has measures to avoid duplicate entries, the arrangement of items in supermarkets, often in multiple locations, can lead to such occurrences. Another important factor for internal validation is quality control. Quality control can be carried out in several manners, depending on the purpose of the study, the time available to work on the database, and the technical capacity of the team. Procedures such as double-entry data tabulation, checking tabulated data in a subset or the entire sample, and performing concordance tests after checking are some of the possible strategies. Therefore, it is important to perform quality control procedures in the database, as well as in each study based on variables of interest. Furthermore, it is important to describe the quality control method adopted as proof of the methodological rigor of the study, as carried out by Aldhirgham et al. (15).

In the 2020 database, only duplicate entries were excluded. Absence of information such as the ingredients list and nutrient contents, among others, was not a reason for excluding foods from the database. Each study based on the database can establish criteria for including or excluding foods according to their variables of interest.

The analysis of quantitative data in food labeling, such as the nutrient declaration in the nutrition information panel, is generally well described in studies analyzing food labels, especially from a statistical point of view. However, the analysis of the ingredient list still seems to be a challenge. The ingredient list is an essential tool for assessing the nutritional quality of packaged foods. However, unlike the nutrient information, there is limited scientific literature focusing on the discussion of the list of ingredients (36, 37).

Although time-consuming, it is fundamental to systematically and individually analyze the ingredient list of all foods included in label studies, rather than solely conducting a search for predefined terms. A thorough analysis may identify potential nonconformities with current regulations in terms of food labels or food product composition. Additionally, it allows the detection of unexpected terms or ingredients. For example, regarding the presence of trans fat in packaged foods, if only terms related to hydrogenated vegetable fat are analyzed, the prevalence may be underestimated. Other ingredients containing trans fat may be listed in the ingredients list, as demonstrated by studies on the subject (26, 38). Two scoping reviews on food labeling studies underscored that the use of predefined terms to identify sweeteners and sugars in ingredient lists could underestimate the prevalence of these components in packaged foods (39, 40).

## 5 Limitations, strengths and perspectives for future research

It is important to note that this type of study may have some limitations, such as high costs, lengthy execution times, and challenges in updating the data (6). One of the challenging points of this approach is the periodic update of data collection, which must be conducted in-person and on-site. As previously mentioned, the cost and time required to collect and tabulate data through photographs is often a limitation. However, unlike other existing methods to collect information from food labels, in-store censuses allow analyzing how the information is available to food consumers at the time of purchase, in a real environment. There is a more complete picture of the reality, reducing the possibility of bias in the choice of samples for study purposes. Additionally, as an indirect result, in-store census methods may contribute to improving the quality of information provided to consumers, becoming relevant in the field of public health and nutrition.

Another limitation of the study is that data collection was carried out in one supermarket. However, in view of the continental dimensions of Brazil, our group chose a supermarket chain that is among the largest chains in the country. While due to this limitation it was not possible to capture regional variations in food availability considering the Brazilian territory, the chain sells a wide diversity of products and brands, 94% of which are sold nationwide, as previously discussed.

As perspectives for future research, it is noteworthy the relevance to building nationwide food labeling databases. This way, the information would be captured from real environments with the labeling information available to food consumers, both in-store and online platforms. It may also include other types of retail formats, such as cash-and-carry stores and—in countries where they are relevant (unlike in Brazil)—discounters. Additionally, it would enable the inclusion of regional variations of foods as well as local brands. Therefore, the packaged foods composition would be monitored from a public health perspective, through the development of cross-sectional and longitudinal studies using labeling information. However, it should be noted, as previously discussed, the challenges associated with building a nationwide food labeling database, especially regarding the costs and efforts needed to maintain and update the data in continental countries such as Brazil.

Additionally, technologies such as artificial intelligence and machine learning are becoming important tools for the construction of national food label databases through in-store census-type methodology, as they can automate time-consuming steps, such as data collection and tabulation. With the use of these tools, it would be possible to update the databases more frequently, covering a greater diversity of foods and brands marketed in the country. This approach could contribute to the monitoring of the composition of packaged foods through information available on labels. These tools may also prove valuable for analyzing qualitative label data, such as ingredients lists, by automating and accelerating the comprehensive assessment of this key labeling element.

## 6 Conclusion

This study underscores the essential role of research on food labeling in guiding the development and reformulation of public policies in food, nutrition, and health. The experience gathered with the process of building 4 food labeling databases in Brazil enabled methodological improvements to the research process, such as a more accurate data collection through food labels photographs, the inclusion of all packaged foods and beverages available for sale in the supermarket and the inclusion of more variables to the analysis. In view of the relevance of the topic, this study proposed a series of methodological recommendations related to data collection, data tabulation and data processing to be carefully considered, designed, and executed during planning, data collection, data tabulation, and database processing. Additionally, the experience permitted the identification of gaps and limitations related to the development of in-store census-type methods, such as the challenges to gather a representative sample of food labels, the difficulties on the transcription of the food labeling data, as well as the costs and efforts needed to maintain and update the data. Furthermore, greater rigor and detail are needed in the methods section of scientific articles on the subject, given that an important premise of the scientific method is replication. This methodological article underscores the importance of raising methodological discussions in the scientific literature to enhance the rigor of in-store census-type approaches.

## Data Availability

The datasets presented in this article are not readily available because the authors do not have permission to share the dataset. Requests to access the datasets should be directed to MK, marianavskraemer@gmail.com.
