# Supplementary material for: Assessing nutritional composition and ingredients of packaged foods in Brazil: an in-store census method for creating a comprehensive food label database
Source: Front Nutr. 2025 Jun 18;12:1568089. doi: 10.3389/fnut.2025.1568089 (PMC12218245; doi:10.3389/fnut.2025.1568089)
Supplement: Supplementary file 1 [file Table_1.docx]

**Supplementary material**

**Table S1.** Studies on food labeling using an in-store census method carried out by the Nutrition in Foodservice Research Centre (NUPPRE) at the Federal University of Santa Catarina, Brazil.

| **Census** | **Theme** | **Publications** |
| --- | --- | --- |
| NUPPRE Brazil 2010 | Analysis of trans fat on food labels | PROENÇA, RPC; SILVEIRA, BM. Recomendações de ingestão e rotulagem de gordura trans em alimentos industrializados brasileiros: análise de documentos oficiais. **Revista de Saúde Pública**, v. 46, p. 923-928, 2012. <https://doi.org/10.1590/S0034-89102012000500020>  SILVEIRA, BM; KLIEMANN, N; SILVA, DP; COLUSSI, CF; PROENÇA, RPC. Availability and price of food products with and without trans fatty acids in food stores around elementary schools in low- and medium-income neighborhoods. **Ecology Food and Nutrition**, v. 52, n.1, p.63-75, 2013. <https://doi.org/10.1080/03670244.2012.705771>  SILVEIRA, BM; GONZALEZ-CHICA, DA; PROENÇA, RPC. Reporting of trans-fat on labels of Brazilian food products. **Public Health Nutrition**, v.16, n.2, p.2146-2153, 2013. <https://doi.org/10.1017/S1368980013000050>  HISSANAGA-HIMELSTEIN, VM; OLIVEIRA, MSV; SILVEIRA, BM; GONZALEZ-CHICA, DA; PROENÇA, RPC; BLOCK, JM. Comparison between Experimentally Determined Total, Saturated and Trans Fat Levels and Levels Reported on the Labels of Cookies and Bread sold in Brazil. **Journal of Food and Nutrition Research**, v. 2, p. 906-913, 2014. <https://doi.org/10.12691/jfnr-2-12-8> |
|  | Analysis of serving sizes and household measures on food labels | MACHADO, PP; KRAEMER, MVS; KLIEMANN, N; GONZALEZ, DA; Proença, RPC. Relação entre porção, medida caseira e presença de gordura trans em rótulos de produtos alimentícios. **O Mundo da Saúde**, v. 37, p. 299-311, 2013.  KLIEMANN, N; VEIROS, MB; GONZALEZ-CHICA, DA; PROENÇA, RPC. Is the serving size and household measure information on labels clear and standardized? Analysis of the labels of processed foods sold in Brazil. **Vigilância Sanitária em Debate: Sociedade, Ciência & Tecnologia**, v. 2, p. 62-68, 2014. <https://doi.org/10.3395/VD.V2I4.445>  KLIEMANN, N; VEIROS, MB; GONZALEZ-CHICA, DA; PROENÇA, RPC. Reference serving sizes for the Brazilian population: An analysis of processed food labels. **Revista de Nutrição**, v. 27, p. 329-341, 2014. <https://doi.org/10.1590/1415-52732014000300007>  KRAEMER, MVS; MACHADO, PP; KLIEMANN, N; GONZALEZ-CHICA, DA; PROENÇA, RPC. The Brazilian population consumes larger serving sizes than those informed on labels. **British Food Journal**, v. 117, p. 719-730, 2015. <https://doi.org/10.1108/BFJ-11-2013-0339>  KLIEMANN, N; KRAEMER, MVS; SILVEIRA, BM; GONZALEZ-CHICA, DA; PROENÇA, RPC. Tamanho da porção e gordura trans: os rótulos dos alimentos brasileiros estão adequados? **DEMETRA**, v. 10, p. 43-60, 2015. <https://doi.org/10.12957/demetra.2015.12981>  KLIEMANN, N; VEIROS, MB; GONZALEZ-CHICA, DA; PROENÇA, RPC. Serving size on nutrition labeling for processed foods sold in Brazil: relationship to energy value. **Revista de Nutrição**, v. 29, p. 741-750, 2016. <https://doi.org/10.1590/1678-98652016000500012>  MACHADO, PP; KRAEMER, MVS; KLIEMANN, N; COLUSSI, CF; VEIROS, MB; PROENÇA, RPC. Serving sizes and energy values on the nutrition labels of regular and diet/light processed and ultra-processed dairy products sold in Brazil. **British Food Journal**, v. 118, p. 1579-1593, 2016. <https://doi.org/10.1108/BFJ-10-2015-0353> |
| NUPPRE Brazil 2011 | Analysis of salt/sodium on food labels | MARTINS, CA; SOUSA, AA; VEIROS, MB; GONZALEZ-CHICA, DA; PROENCA, RPC. Sodium content and labelling of processed and ultra-processed food products marketed in Brazil. **Public Health Nutrition**, v. 18, p. 1206-1214, 2015. <https://doi.org/10.1017/S1368980014001736>  NISHIDA, W; FERNANDES, AC; VEIROS, MB; GONZALEZ-CHICA, DA; PROENÇA, RPC. A comparison of sodium contents on nutrition information labels of foods with and without nutrition claims marketed in Brazil. **British Food Journal**, v. 118, p. 1594-1609, 2016. <https://doi.org/10.1108/BFJ-09-2015-0325>  KRAEMER, MVS; OLIVEIRA, RC; GONZALEZ-CHICA, DA; PROENÇA, RPC. Sodium content on processed food labels for snacks consumed by Brazilian children and adolescents. **Public Health Nutrition**, v. 19, s 6, p. 967-975, 2016. <https://doi.org/10.1017/S1368980015001718> |
| NUPPRE Brazil 2013 | Analysis of nutrition claims on the labels of foods targeted at children | RODRIGUES, VM; RAYNER, M; FERNANDES, AC; OLIVEIRA, RC; PROENÇA, RPC; FIATES, GMR. Comparison of the nutritional content of products, with and without nutrient claims, targeted at children in Brazil. **British Journal of Nutrition**, v. 115, p. 2047-2056, 2016. <https://doi.org/10.1017/S0007114516001021>  ZUCCHI, ND; FIATES, GMR. Analysis of the presence of nutrient claims on labels of ultra-processed foods directed at children and of the perception of kids on such claims**. Revista de Nutrição**, v. 29, p. 821-832, 2016. <https://doi.org/10.1590/1678-98652016000600007> |
|  | Analysis of the nutritional quality of foods targeted at children | RODRIGUES, VM; RAYNER, M; FERNANDES, AC; OLIVEIRA, RC; PROENÇA, RPC; FIATES, GMR. Nutritional quality of packaged foods targeted at children in Brazil: which ones should be eligible to bear nutrient claims? **International Journal of Obesity**, p. 1-12, 2016.  <https://doi.org/10.1038/ijo.2016.167>  MACHADO, M. L.; RODRIGUES, V. M.; NASCIMENTO, A. B.; DEAN, M.; FIATES, G. M. R. Nutritional composition of Brazilian food products marketed to children. **Nutrients**, v. 11, n. 6, p. 1214-1214, 2019.  <https://doi.org/10.3390/nu11061214> |
|  | Analysis of information on transgenic ingredients on food labels | CORTESE, RDM; MARTINELLI, SS; FABRI, RF; PROENÇA, RPC; CAVALLI, SB. A label survey to identify ingredients potentially containing GM organisms to estimate intake exposure in Brazil. **Public Health Nutrition**, v. 21, p. 2698-2713, 2018. <https://doi.org/10.1017/S1368980018001350> |
|  | Analysis of added sugars on food labels | SCAPIN, T; FERNANDES, AC; PROENCA, RPC. Added sugars: definitions, classifications, metabolism and health implications. **Revista de Nutrição**, v. 30, p. 1-12, 2017. <https://doi.org/10.1590/1678-98652017000500011>  SCAPIN, T; FERNANDES, AC; PROENCA, RPC. Use of added sugars in packaged foods sold in Brazil. **Public Health Nutrition**, p. 1-7, 2018. <https://doi.org/10.1017/S1368980018002148>  SCAPIN, T; CHUN YU LOUIE, J; PETTIGREW, S; NEAL, B; RODRIGUES, VM; FERNANDES, AC ; BERNARDO, GL; UGGIONI, PL; PROENÇA, RPC. The adaptation, validation, and application of a methodology for estimating the added sugar content of packaged food products when total and added sugar labels are not mandatory. **Food Research International**, p. 110329-43, 2021. <https://doi.org/10.1016/j.foodres.2021.110329>  SCAPIN, T; FERNANDES, AC; CURIONI, CC; PETTIGREW, S; NEAL, B; COYLE, DH; RODRIGUES, VM; BERNARDO, GL; UGGIONI, PL; ROENÇA, RPC. Influence of sugar label formats on consumer understanding and amount of sugar in food choices: a systematic review and meta-analyses. **Nutrition Reviews,** v. 79, p. 788-801, 2021. <https://doi.org/10.1093/nutrit/nuaa108>  SCAPIN, T.; FERNANDES, A. C.; SHAHID, M.; Pettigrew, S.; KHANDPUR, N. ; BERNARDO, G. L. ; UGGIONI, Paula Lazzarin; Proença, Rossana Pacheco da Costa . Consumers' response to sugar label formats in packaged foods: a multi-methods study in Brazil. **Frontiers in Nutrition**, v. 9, p. 896784, 2022. <https://doi.org/10.3389/fnut.2022.896784>  SANTANA, IP; SCAPIN, T; RODRIGUES, VM; BERNARDO, GL; UGGIONI, PL; FERNANDES, AC; PROENÇA, RPC. University Students' Knowledge and Perceptions About Concepts, Recommendations, and Health Effects of Added Sugars. **Frontiers in Nutrition**, v. 9, p. 1-7, 2022. <https://doi.org/10.3389/fnut.2022.896895> |
|  | Analysis of sweeteners on foods labels | FIGUEIREDO, LS; SCAPIN, T; FERNANDES, AC; PROENÇA, RPC. Where are the low-calorie sweeteners? An analysis of the presence and types of low-calorie sweeteners in packaged foods sold in Brazil from food labelling. **Public Health Nutrition**, v. 21, p. 447-453, 2017. <https://doi.org/10.1017/S136898001700283X>  SCAPIN, T; FERNANDES, AC; COYLE, DH; PETTIGREW, S; FIGUEIREDO, LS; GERALDO, APG; PROENÇA, RPC. Packaged foods containing non-nutritive sweeteners also have high added sugar content: A Brazilian survey. **Journal of Food Composition and Analysis**, v. 111, p. 104626, 2022. <https://doi.org/10.1016/j.jfca.2022.104626> |
|  | Analysis of additives on food labels with "homemade" terms | KANEMATSU, LRA; MULLER, J; FABRI, RF; SCAPIN, T; COLUSSI, CF; PROENÇA, RPC; FERNANDES, AC ; BERNARDO, GL; UGGIONI, PL. Do foods products labelled 'home-made' contain fewer additives? A Brazilian survey. **Journal of Food Products Marketing**, v. 1, p. 1-12, 2020. <https://doi.org/10.1080/10454446.2020.1811185> |
|  | Analysis of nutritional quality and the use of the term "whole" on food labels | BATTI, EAB; NASCIMENTO, AB; GERALDO, APG; FERNANDES, AC; BERNARDO, GL; PROENÇA, RPC; UGGIONI, PL Use of the term whole grain on the label of processed and ultra-processed foods based on cereals and pseudocereals in Brazil. **Frontiers in Nutrition**, v. 9, p. 1-11, 2022. <https://doi.org/10.3389/fnut.2022.875913> |
|  | Analysis of trans fat on food labels | BARROS, BIV; PROENÇA, RPC; KLIEMANN, N; HILLESHEIN, D; SOUZA, AA; CEMBRANEL, F.; BERNARDO, GL; UGGIONI, PL; FERNANDES, AC. Trans-fat labeling in packaged foods sold in Brazil before and after changes in regulatory criteria for trans fat-free claims on food labels. **Frontiers in Nutrition**, v. 9, p. 1-15, 2022. <https://doi.org/10.3389/fnut.2022.868341> |
|  | Analysis of vitamins and minerals on food labels targeted at children | MARTINS, AC; KRAEMER, MVS; RODRIGUES, VM; HINNIG PF; FERNANDES, AC; BERNARDO, GL; PROENÇA, RPC; UGGIONI, PL. Market-driven fortification of vitamins and minerals in packaged foods targeted at children in Brazil. **Nutrition Bulletin**, v. 49, p. 209-219, 2024.  <https://doi.org/10.1111/nbu.12676> |
| NUPPRE/FoodSwitch Brazil 2020 | Analysis of additives in foods targeted at children | KRAEMER, MVS; FERNANDES, AC; CHADAD, MCC; UGGIONI, PL; RODRIGUES, VM; BERNARDO, GL; PROENÇA, RPC. Food additives in childhood: a review on consumption and health consequences. **Revista Saúde Pública**, v. 56, p. 1-22, 2022. <https://doi.org/10.11606/s1518-8787.2022056004060>  KRAEMER, MVS. ; FERNANDES, AC; Chaddad, MCC; UGGIONI, PL; BERNARDO, GL; PROENÇA, RPC. Is the List of Ingredients a Source of Nutrition and Health Information in Food Labeling? A Scoping Review. **Nutrients**, v. 15, p. 4513, 2023. <https://doi.org/10.3390/nu15214513>  KRAEMER, MVS.; FERNANDES, AC; ARES, G; CHADDAD, MCC; PETTIGREW, S; SCAPIN, T; UGGIONI, PL; BERNARDO, GL; PROENÇA, RPC. Infant and children's exposure to food additives: an assessment of a comprehensive packaged food database. **Journal of Food Composition and Analysis**, v. 134, p. 106473, 2024. <https://doi.org/10.1016/j.jfca.2024.106473> |
|  | Analysis of industrial trans fat and substitutes on food labels | BARROS, B.V.; KRAEMER, M.V.S.; MILANO, E.; BERNARDO, G. L.; CHADDAD, M. C. C.; UGGIONI, P. L.; PROENÇA, R. P. C.; FERNANDES, A. C. Substitutes for industrial trans fats in packaged foods: a scoping review. **Nutrition Reviews,** 2025. Online ahead of print. <https://doi.org/10.1093/nutrit/nuae194>.  Manuscript in preparation: cross-sectional and longitudinal analysis of trans fats and their substitutes on foods labels |
|  | Conceptualization and analysis of sweeteners on food labels | MILANO, E.; GERALDO, A.P.G.; KRAEMER, M. V. S.; ARES, G.; BARROS, B.V.; CHADDAD, M. C. C.; FERNANDES, A. C.; BERNARDO, G. L.; UGGIONI, P. L.; PROENÇA, R. P. C. Declaration of sweeteners on food labels: a scoping review of methodological issues and prevalence. **Nutrition Reviews,** 2025. Online ahead of print. <https://doi.org/10.1093/nutrit/nuae175>.  Manuscript in preparation: analysis of sweeteners on food labels |
|  | Analysis of free sugars from fruits on food labels | PADOVAN, M.; ARES, G.; SCAPIN, T.; KRAEMER, M. V. S.; CHADDAD, M. C. C.; FERNANDES, A. C.; BERNARDO, G. L.; UGGIONI, P. L.; PETTIGREW, S.; PROENÇA, R. P. C. Declaration of free sugars from fruits on food labels: a scoping review. **British Food Journal,** v. 126, p. 4342-4356, 2024. <https://doi.org/10.1108/BFJ-05-2024-0547>  Manuscript in preparation: analysis of free sugars from fruits on food labels |
|  | Analysis of sugars in foods targeted at children | Manuscript in preparation |
|  | Comparison between ingredients of packaged foods with the claim "homemade" and their counterparts in traditional culinary recipes | Manuscript in preparation |
|  | Analysis of additives on food labels with the claims "homemade," "traditional," and their analogs | Manuscript in preparation |
|  | Comparison of the nutritional composition of foods formulated with whole grains and pseudocereals sold in Brazil in 2013 and 2020 | Manuscript in preparation |
| Other studies adopting an in-store census method | Evaluation of front-of-pack labeling models and adequacy of serving sizes in packaged foods sold in Brazil | KLIEMANN, N; KRAEMER, MVS; SCAPIN, T; RODRIGUES, VM; FERNANDES, AC; BERNARDO, GL; UGGIONI, PL; PROENÇA, RPC. Serving size and nutrition labelling: implications for nutrition information and nutrition claims on packaged foods. **Nutrients,** v. 10, p. 891-904, 2018. <https://doi.org/10.3390/nu10070891>  MAZZONETTO, AC; FERNANDES, AC; SOUZA, AD; RODRIGUES, VM; SCAPIN, T; UGGIONI, PL; VEIROS, M. B. ; BERNARDO, GL; PROENÇA, R.P.C. Front-of-pack nutrition labels: perceptions and preferences of Brazilian adult consumers. **British Food Journal,** Vol. 124 No. 11, pp. 3888-3906, 2022. <https://doi.org/10.1108/BFJ-05-2021-0588> |
|  | Definition and use of the term "clean label" in the labeling of packaged foods: a scoping review | Manuscript in preparation |
